# Supplementary material for: Evaluating the CRP Interactome: Insights into Possible Novel Roles in Cellular Signaling and Tumorigenicity
Source: Curr Issues Mol Biol. 2025 Nov 28;47(12):1003. doi: 10.3390/cimb47121003 (PMC12732217; doi:10.3390/cimb47121003)
Supplement: Supplementary file 1 [file cimb-47-01003-s001.zip › cimb-3979309-supplementary.pdf]

Gene ID	Protein Name	General Function	Experimental Evidence	Reference
**ADAM9**	ADAM metallopeptidase domain 9	Proteolysis and cell–cell interaction a metalloproteinase	Affinity Capture Mass Spec	23
**AGPAT1**	1-acylglycerol-3-phosphate O-acyltransferase 1	Enzyme in phospholipid biosynthesis; converts lysophosphatidic acid to phosphatidic acid	Affinity Capture Mass Spec	23
**AGRN**	Agrin	Heparan sulfate proteoglycan important for the development of neuromuscular development, also Involved in membrane integrity, CNS synapse function	Affinity Capture Mass Spec	23<br>26
**APCS**	Amyloid P component, serum	Component of amyloid deposits and involved in immune regulation	Reconstituted Complex	27
**B4GALT1**	Beta 1,4-galactosyltransferase 1	Transfers galactose to GlcNAc residues in glycoproteins and glycolipids	Affinity Capture Mass Spec	23
**B4GALT3**	Beta-1,4-Galactosyltransferase 3	Transfers UDP-galactose to beta 1,4 linkages (e.g., GlcNAc); Biosynthesis of aminoglycans tetrasaccharide linkage region of proteoglycans	Affinity Capture Mass Spec	23<br>26
**B4GALT4**	Beta-1,4-Galactosyltransferase 3	Transfers UDP-galactose to beta 1,4 linkages; involved in proteoglycan biosynthesis of aminoglycans tetrasaccharide linkage region of proteoglycans	Affinity Capture Mass Spec	23
**B4GALT5**	Beta-1,4-Galactosyltransferase 5	Transfers UDP-galactose to beta 1,4 linkages (e.g., GlcNAc); Biosynthesis of aminoglycans tetrasaccharide linkage region of proteoglycans	Affinity Capture Mass Spec	23<br>26
**B4GALT7**	Beta-1,4-Galactosyltransferase 7	Transfers UDP-galactose to beta 1,4 linkages (e.g., GlcNAc); Biosynthesis of aminoglycans tetrasaccharide linkage region of proteoglycans	Affinity Capture Mass Spec	23<br>26
**BMP4**	Bone morphogenetic protein 4	Regulates development which includes bone and cartilage formation	Affinity Capture Mass Spec	23
**C1GALT1**	Core 1 synthase, glycoprotein-N-acetylgalactosamine 3-beta-galactosyltransferase 1	Synthesizes core 1 O-glycan structures in mucin type glycoproteins	Affinity Capture Mass Spec	26
**C1QA**	Complement component 1, q subcomponent, A chain	Initiates the complement pathway for immune defense	Reconstituted Complex	28
**CA11**	Carbonic anhydrase XI	Catalyzes reversible hydration of carbon dioxide and involved in brain function	Affinity Capture Mass Spec	26
**CFH**	Complement factor H	Regulates complement activation on self-cells to prevent damage	Reconstituted Complex	35
**CHST12**	Carbohydrate (chondroitin 4) sulfotransferase 12	Transfers sulfate groups to position 4 of N-acetylgalactosamine in chondroitin	Affinity Capture Mass Spec	23<br>26

**CHST14**	Carbohydrate (N-acetylgalactosamine 4-0) sulfotransferase 14	Transfers sulfate to N-acetylgalactosamine residues	Affinity Capture Mass Spec	23
**CHST3**	Carbohydrate (chondroitin 6) sulfotransferase 3	Transfers sulfate groups to position 6 of N-acetylgalactosamine in chondroitin	Affinity Capture Mass Spec	23<br>26
**CHST6**	Carbohydrate (N-acetylglucosamine 6-O) sulfotransferase 6	Involved in keratan sulfate biosynthesis	Affinity Capture Mass Spec	23
**CNTNAP1**	Contactin associated protein 3	Formation of the paranodal junction in myelinated nerves	Affinity Capture Mass Spec	23
**CNTNAP3**	Contactin associated protein-like 3	Cell adhesion and communication in the nervous system	Affinity Capture Mass Spec	23<br>26
**CNTNAP3B**	Contactin associated protein-like 3B	Might function in neural development	Affinity Capture Mass Spec	23
**COL18A1**	Collagen type XVIII alpha 1	Structural component of basement membranes	Affinity Capture Mass Spec	23<br>26
**COL4A2**	Collagen, type IV, alpha 2	Structural component of basement membranes	Affinity Capture Mass Spec	23
**CSPG4**	Chondroitin sulfate proteoglycan 4	Involved in cell adhesion, migration and proliferation	Affinity Capture Mass Spec	23
**DGCR14**	DiGeorge syndrome critical region gene 14	mRNA splicing and associated with DiGeorge syndrome	Two-hybrid	29
**EXT1**	Exostosin glycosyltransferase 1	Heparan sulfate biosynthesis; glycosyltransferase	Affinity Capture Mass Spec	23<br>26
**FN1**	Fibronectin 1	Major extracellular matrix protein involved in cell adhesion and wound healing	Reconstituted Complex	20
**GDF11**	Growth differentiation factor 11	Regulates development and differentiation; member of TGF-beta superfamily	Affinity Capture Mass Spec	23<br>26
**GLUD1**	Glutamate dehydrogenase 1	Enzyme which converts glutamate to alpha-ketoglutarate	Two-hybrid	29
**GMPPA**	GDP-mannose pyrophosphorylase A	Enzyme involved in the biosynthesis of GDP-mannose	Two-hybrid	30

**HIST1H1A**	Histone cluster 1,H1a	DNA package in chromatin	Reconstituted Complex	31
**HIST2H2AC**	Histone cluster 2,H2ac	Main component of nucleosome and involved in DNA compaction	Reconstituted Complex	31
**HS3ST1**	Heparan sulfate (glucosamine) 3-O-sulfotransferase 1	Important role in the biosynthesis of heparan sulfate adding 3-O-sulfate groups to certain glucosamine residues in heparan sulfate chains	Affinity Capture Mass Spec	23
**HS6ST1**	Heparan sulfate 6-O-sulfotransferase 1	Catalyzes sulfation in heparan sulfate biosynthesis	Affinity Capture Mass Spec	23<br>26
**HSPA5**	Heat shock 70kDa protein 5	Important role in protein quality control, ER stress response and cell survival	Proximity Label Mass Spec	32
**IDUA**	Iduronidase, alpha-L-	important role in the breakdown of glycosaminoglycans	Affinity Capture Mass Spec	23
**IMPAD1**	Inositol monophosphatase domain containing 1	Hydrolyzes phosphoadenosine phosphate into AMP, helping glycosaminoglycan sulfation and skeletal development	Affinity Capture Mass Spec	26
**LACTB**	Serine beta-lactamase	Forming intermembrane filaments and regulating mitochondrial lipid metabolism.	Affinity Capture Mass Spec	23
**LAMA1**	Laminin, alpha 1	Major component of the basal lamina; involved in cell adhesion and differentiation	Affinity Capture Mass Spec	23<br>26
**LAMA5**	Laminin, alpha 5	Major component of the basal lamina; involved in tissue organization and cell adhesion	Affinity Capture Mass Spec	23<br>26
**LAMB1**	Laminin, beta 1	Component of laminin proteins; involved in cell adhesion and migration	Affinity Capture Mass Spec	23<br>26
**LAMB2**	Laminin, beta 2	Supports basement membrane structure; critical for neuromuscular synapse formation	Affinity Capture Mass Spec	23<br>26
**LAMC1**	Laminin gamma-1	Promotes osteogenic differentiation and inhibiting adipogenesis implicated in tumor invasion and metastasis	Affinity Capture Mass Spec	23
**LAMC3**	Laminin gamma 3	Glycoprotein in extracellular matrix mediating cell adhesion, migration and organizational development	Two-hybrid	29
**LRRC24**	Leucine rich repeat containing 24	A single transmembrane protein made up of LRR and Ig domains mainly expressed in the brain	Affinity Capture Mass Spec	23

**MAPK1**	Mitogen-activated protein kinase 1	Transduces signals from cell surface to the nucleus, cell growth, division, differentiation and survival	Two-hybrid	29
**MAPK3**	Mitogen-activated protein kinase 3	Transduces extracellular signals into intracellular responses to growth and development	Two-hybrid	33
**MAPK8IP2**	Mitogen-activated protein kinase 8 interacting protein 2	Neuronal development, signal coordination and apoptosis regulation, mainly organize and regulate MAPK signaling	Two-hybrid	34
**NDST2**	N-deacetylase/N-sulfotransferase 2	Bifunctional golgi enzyme essential for heparan sulfate biosynthesis removing acetyl groups adding sulfate to glucosamine	Affinity Capture Mass Spec	26
**NXPE3**	Neurexophilin and PC-esterase domain family, member 3	Role in nervous system development and signaling	Affinity Capture Mass Spec	23<br>26
**PDF**	Peptide deformylase	Removes formyl group from nascent mitochondrial proteins	Affinity Capture Mass Spec	23<br>26
**PDIA5**	Protein disulfide isomerase family A 5	Protein folding in the endoplasmic reticulum, disulfide bond isomerase	Affinity Capture Mass Spec	23
**PRSS23**	Protease, serine, 23	May be involved in tissue remodeling through proteolytic activity	Affinity Capture Mass Spec	23<br>26
**PTPRS**	Protein tyrosine phosphatase, receptor type S	Cell signaling regulator; involved in neuronal development	Affinity Capture Mass Spec	23<br>26
**QSOX1**	Quiescin Q6 sulfhydryl oxidase 1	Involved in disulfide bond formation in proteins	Affinity Capture Mass Spec	23<br>26
**RPL13A**	Ribosomal protein L13a	Structural component of the 60S large subunit; protein synthesis and regulatory functions	Two-hybrid	29
**RPL23**	Ribosomal protein L23	Structural component of the 60S ribosomal subunit; protein synthesis	Affinity Capture Mass Spec	23<br>26
**RPL26L1**	Ribosomal protein L26-like 1	Considered to be ribosomal protein involved in translation	Affinity Capture Mass Spec	23<br>26
**SNRNP70**	Small nuclear ribonucleoprotein 70kDa (U1)	Important for pre-mRNA splicing removing introns from precursor mRNA	Affinity Capture-Western	31
**SPTB**	Spectrin beta (erythrocitic)	Responsible for disulfide bond formation in some proteins	Affinity Capture Mass Spec	23<br>26

**ST3GAL4**	ST3 beta-galactoside alpha-2,3-sialyltransferase 4	Adds sialic acid to galactose-containing substrates in glycoproteins	Affinity Capture Mass Spec	23<br>26
**ST8SIA6**	ST3 alpha-n-acetyl-neuraminide alpha-2,8-sialyltransferase 6	Adds sialic acid residues to glycoproteins and glycolipids	Affinity Capture Mass Spec	23
**STC2**	Stanniocalcin 2	Involved in calcium/phosphate homeostasis and cellular stress response	Affinity Capture Mass Spec	23<br>26
**STRBP**	Spermatid perinuclear RNA binding protein	Involved in RNA regulation mainly in the nervous system and male germ cell	Affinity Capture Mass Spec	26
**TGFB1**	Transforming growth factor beta 1	Multifunctional cytokine important part in the central regulatory in development, immune response and tissue repair	Affinity Capture Mass Spec	23
**TIMP3**	TIMP metallopeptidase inhibitor 3	Inhibits metalloproteinases; involved in extracellular matrix regulation	Affinity Capture Mass Spec	23<br>26
**TMEM2**	Transmembrane protein 2	May play a role in cell signaling or structural integrity	Affinity Capture Mass Spec	23<br>26
**TMEM59**	Transmembrane protein 59	Type 1 membrane protein involved in cellular signaling and membrane trafficking, influences apoptosis and autophagy	Affinity Capture Mass Spec	23
**UBE3A**	Ubiquitin protein ligase E3A	Important role in protein degradation, neuronal function and neurodevelopment	Two-hybrid	35
**UBR3**	Ubiquitin protein ligase E3 component n-recognin 3	Considered to be E3 ligase; regulate protein turnover through ubiquitination	Affinity Capture Mass Spec	23<br>26
**VGF**	Nerve growth factor inducible protein	Secreted protein generating peptides and regulating metabolism, reproduction, pain, as well as emotional behavior.	Affinity Capture Mass Spec	23

Supplementary Table 1. C-Reactive Protein (CRP) BioGRID Protein–Protein Interaction (PPI) Dataset. Full BioGRID (thebiogrid.org) dataset of all the potential CRP protein–protein interactions (PPIs) including gene name, full protein name, general function, experimental method for identification, and related reference. Database was queried and gene list was curated on May 1st, 2025. References listed can be found in the *References* section of the manuscript.

Gene Ontology: Molecular Function	# Genes in Overlap (k)	FDR q-value	CRP PPIs
Structural Molecule Activity	17	3.43E-11	AGRN, COL18A1, COL4A2, FN1, H1-1, H2AC20, LAMA1, LAMA5, LAMB1, LAMB2, LAMC1, LAMC3, MAPK8IP2, RPL13A, RPL23, RPL26L1, SPTB
Sulfotransferase Activity	7	3.49E-11	CHST12, CHST14, CHST3, CHST6, HS3ST1, HS6ST1, NDST2
Extracellular Matrix Structual Constituent	9	6.14E-09	AGRN, COL18A1, COL4A2, FN1, LAMA1, LAMA5, LAMB1, LAMB2, LAMC1
Galactosyltransferase Activity	6	8.42E-09	B4GALT1, B4GALT3, B4GALT4, B4GALT5, B4GALT7, C1GALT1
Transferase Activity Transferring Sulphur Containing Groups	7	1.08E-08	CHST12, CHST14, CHST3, CHST6, H3ST1, HS6ST1, NDST2
N-Aceyllactosamine Synthase Activity	4	1.08E-08	B4GALT1, B4GALT3, B4GALT4, B4GALT5
Glycotransferase Activity	9	2.50E-07	B4GALT1, B4GALT3, B4GALT4, B4GALT5, B4GALT7, C1GALT1, EXT1, ST3GAL4, ST8SIA6
Hexosyltransferase Activity	7	1.14E-05	B4GALT1, B4GALT3, B4GALT4, B4GALT5, B4GALT7, C1GALT1, EXT1
UDP Glycotransferase Activity	6	3.68E-05	B4GALT1, B4GALT3, B4GALT4, B4GALT5, B4GALT7, EXT1
Sulfur Compound Binding	7	6.64E-05	AGRN, BMP4, CFH, CHST12, FN1, H1-1, PTPRS

Supplementary Table 2. PPIs with C-Reactive Protein are Enriched in Multiple Metabolic Processes. Gene Set Enrichment Analysis (GSEA) was performed using the Molecular Signature Database (gsea.org, accessed 05/01/25). All 71 proteins with protein–protein interactions (PPIs) curated by BioGrid (thebiogrid.org, accessed 05/01/25) from published data were used and the "Gene Ontology Molecular Function" database was queried. Signaling pathways are indicated with the number of proteins identified per pathway, with the false-discovery rate (FDR) q-value and the specific PPIs per signaling pathway identified.

Hallmarks of Cancer Pathways	# Genes in Overlap (k)	FDR q-value	CRP PPIs
Glycolysis	12	4.15E-14	AGRN, B4GALT1, B4GALT4, B4GALT7, CHST6, CHST12, EXT1, HSPA5, GMPPA, IDUA, QSOX1, STC2
Epithelial–Mesenchymal Transition	7	1.17E-06	COL4A2, FN1, LAMA1, LAMC1, QSOX, TGFB1, TIMP3
Coagulation	6	2.09E-06	ADAM9, C1QA, CFH, FN1, PRSS23, TIMP3
Complement	6	1.11E-05	ADAM9, C1QA, CHF, COL4A2, FN1, HSPA5
Hypoxia	6	1.11E-05	CHST3, HS3ST1, HSPA5, EXT1, NDST2, STC2
Myogenesis	4	2.99E-03	AGRN, COL4A2, STC2, TGFB1
Unfolded Protein Response	3	6.52E-03	HSPA5, PDIA5, STC2
Estrogen Response	3	2.88E-02	B4GALT1, PRSS23, STC2

Supplementary Table 3. PPIs with C-Reactive Protein are Enriched in Key Tumorigenic Signaling Processes. Gene Set Enrichment Analysis (GSEA) was performed using the Molecular Signature Database (gsea.org, accessed 05/01/25). All 71 proteins with protein–protein interactions (PPIs) curated by BioGrid (thebiogrid.org, accessed 05/01/25) from published data were used and the "Hallmarks of Cancer" database was queried. Signaling pathways are indicated with the number of proteins identified per pathway, with the false-discovery rate (FDR) q-value and the specific PPIs per signaling pathway identified.

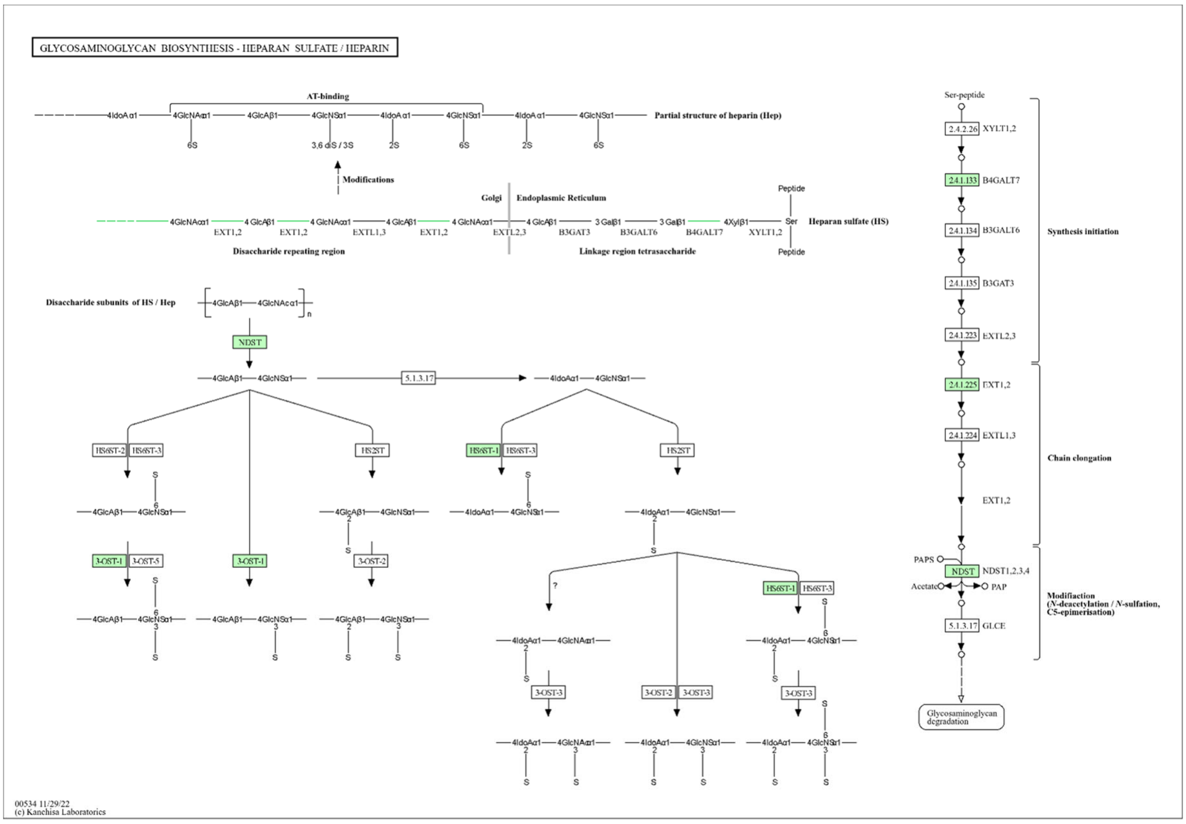

Supplementary Figure 1. CRP PPIs in KEGG Heparan Sulfate Biosynthesis. KEGG Map of the Glycosaminoglycan Heparan Sulfate Biosynthesis pathway referencing the CRP interactions with B4GALT7, EXT1, HS3ST1, HS6ST1, and NDST2. PPIs are demarked in green. Data was obtained using ShinyGO 0.82 (bioinformatics.sdstate.edu/go, accessed 05/01/25).

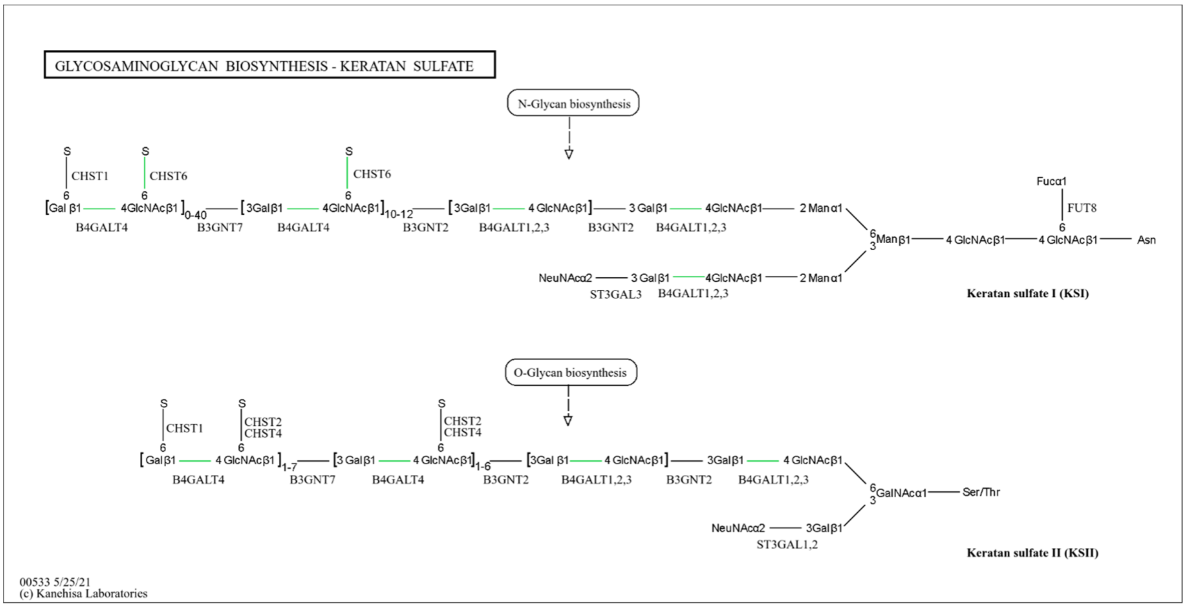

Supplementary Figure 2. CRP PPIs in KEGG Keratan Sulfate Biosynthesis. KEGG Map of the Glycosaminoglycan Keratan Sulfate Biosynthesis pathway referencing the CRP interactions with B4GALT1, B4GALT3, B4GALT4, and CHST6. PPIs are demarked in green. Data was obtained using ShinyGO 0.82 (bioinformatics.sdstate.edu/go, accessed 05/01/25).

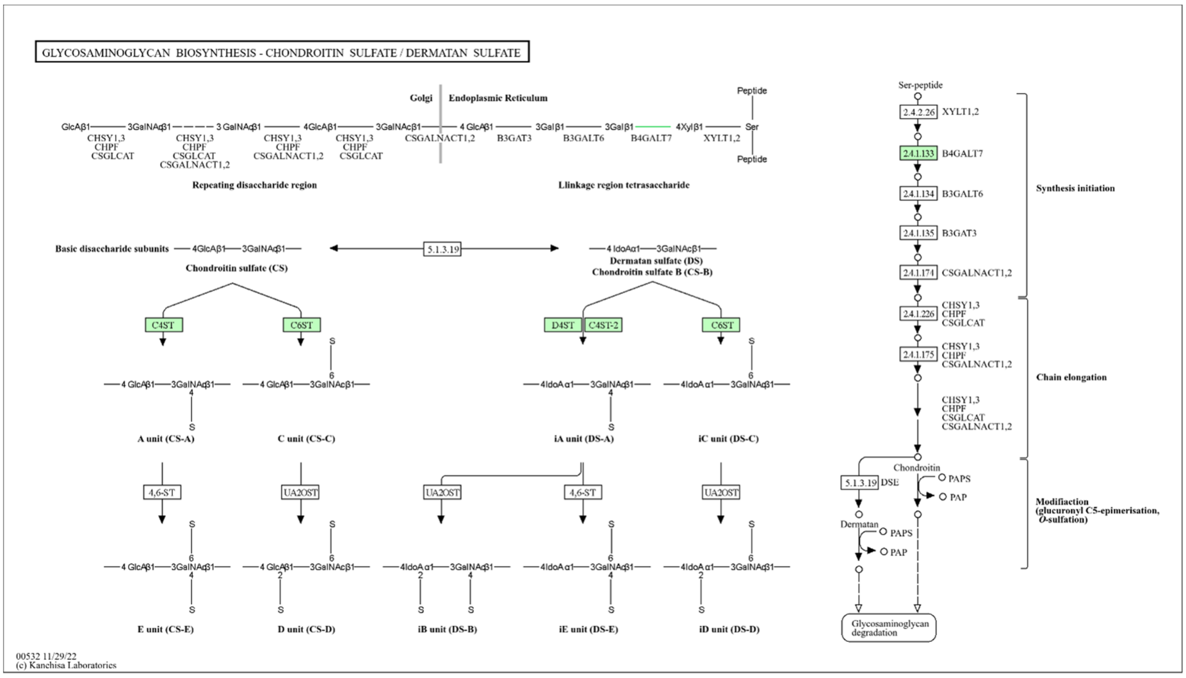

Supplementary Figure 3. CRP PPIs in KEGG Chondroitin Sulfate Biosynthesis. KEGG Map of the Glycosaminoglycan Keratan Sulfate Biosynthesis pathway referencing the CRP interactions with B4GALT7, CHST3, CHST12, and CHST14. PPIs are demarked in green. Data was obtained using ShinyGO 0.82 (bioinformatics.sdstate.edu/go, accessed 05/01/25).

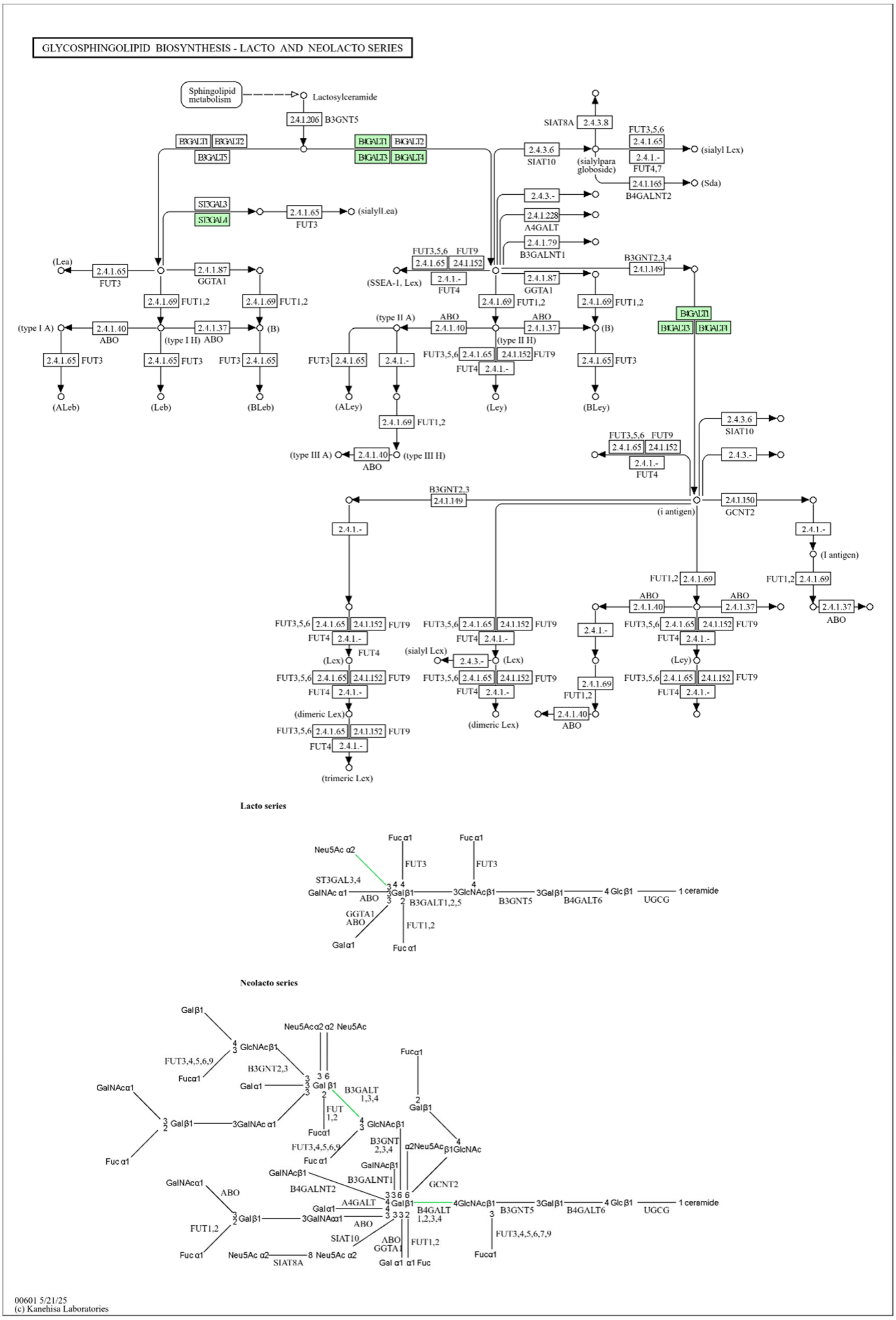

Supplementary Figure 4. CRP PPIs in KEGG Glycosphingolipid Biosynthesis. KEGG Map of the Glycosphingolipid Biosynthesis Lacto/Neolacto pathway referencing the CRP interactions with B4GALT1, B4GALT3, B4GALT4, and ST3GAL4. PPIs are demarked in green. Data was obtained using ShinyGO 0.82 (bioinformatics.sdstate.edu/go, accessed 05/01/25).

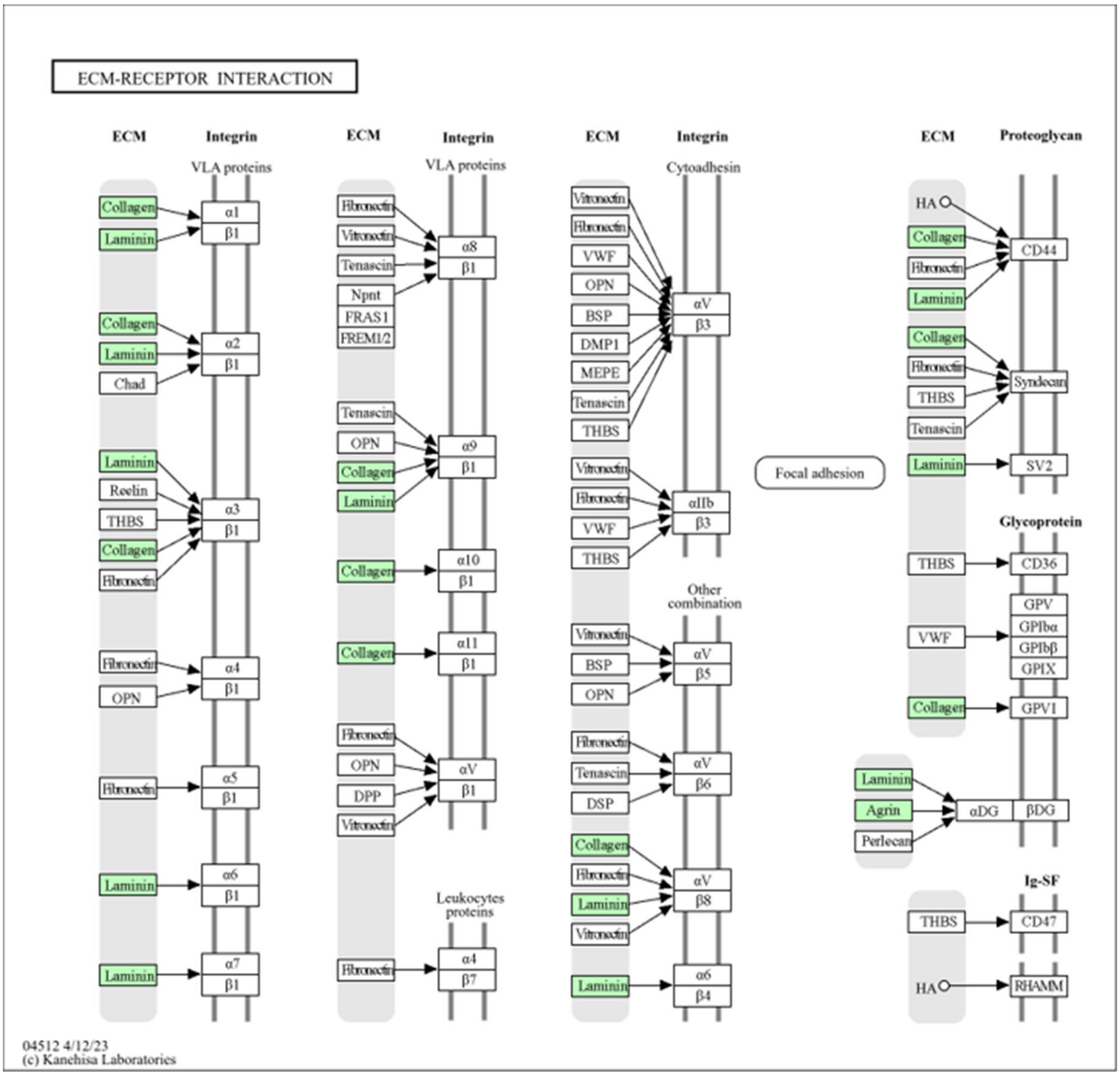

Supplementary Figure 5. CRP PPIs in KEGG ECM–Receptor Interactions. KEGG Map of the ECM–Receptor pathway referencing the CRP interactions with AGRN, COL4A2, FN1, LAMA1, LAMA5, LAMB1, LAMB2, LAMC1, and LAMC3. PPIs are demarked in green. Data was obtained using ShinyGO 0.82 (bioinformatics.sdstate.edu/go, accessed 05/01/25).

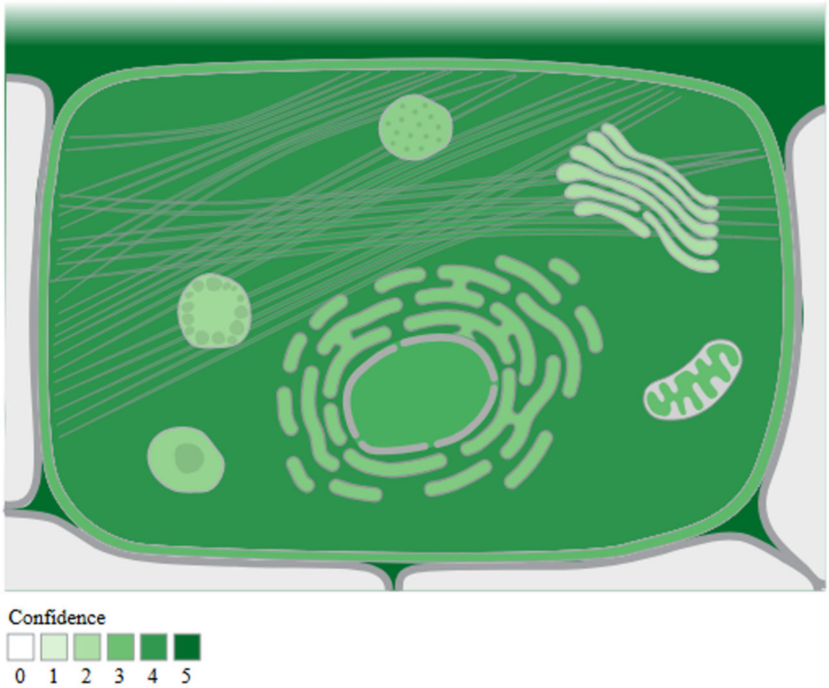

Name	Source	Evidence	Confidence
Extracellular region	UniProtKB	CURATED	★★★★★
Extracellular region	UniProtKB	NAS	★★★★★
Extracellular space	BHF-UCL	IDA	★★★★☆

Predictions from Text Mining

Name	Z-score	Confidence
Troponin complex	8.1	★★★★☆
Extracellular ferritin complex	7.8	★★★★☆
Calprotectin complex	7.6	★★★★☆
Nucleus	6.9	★★★★☆
Pituitary gonadotropin complex	6.8	★★★★☆
Plasma membrane	6.3	★★★★☆
Very-low-density lipoprotein particle	6.3	★★★★☆
Mitochondrion	6.0	★★★★☆

Supplementary Figure 6. CRP Intracellular and Extracellular Localization. Predictive model of CRP localizations obtained from the COMPARTMENTS subcellular localization database (compartments.jensenlab.org, accessed 05/01/25) with direct curated evidence (e.g., via UniProtKB) or predicted via text mining. Potential subcellular localizations of CRP are demarked in green, with dark green representing high confidence, light green representing less confidence, and gray/white representing it is not predicted to occur in that location.
